# Semen quality as a potential susceptibility indicator to SARS-CoV-2 insults in polluted areas

**DOI:** 10.1007/s11356-021-14579-x

**Published:** 2021-05-29

**Authors:** Luigi Montano, Francesco Donato, Pietro Massimiliano Bianco, Gennaro Lettieri, Antonino Guglielmino, Oriana Motta, Ian Marc Bonapace, Marina Piscopo

**Affiliations:** 1 Andrology Unit, EcoFoodFertility Project, Coordination Unit, Local Health Authority (ASL) Salerno, Oliveto Citra, Via M. Clemente, 84020 Oliveto Citra, SA Italy; 2 Department of Medical and Surgical Specialties Radiological Sciences and Public Health, Unit of Hygiene, Epidemiology, and Public Health, University of Brescia, Brescia, Italy; 3 ISPRA, Italian Institute for Environmental Protection and Research, Via Vitaliano Brancati 60, 00144 Rome, Italy; 4 Department of Biology, University of Naples Federico II, Via Cinthia 21, 80126 Napoli, Italy; 5 Unit of Reproductive Medicine (UMR) Catania, Catania, Italy; 6 Department of Medicine, Surgery and Dentistry, University of Salerno, Fisciano, Italy; 7 Department of Biotechnology and Life Sciences, University of Insubria (VA), Varese, Italy

**Keywords:** Air pollution, COVID-19, Semen quality, Environmental marker, Health marker, Oxidative stress, SARS-CoV-2

## Abstract

The epidemic of the new severe acute respiratory syndrome coronavirus 2 (SARS-CoV-2) has impacted worldwide with its infectious spread and mortality rate. Thousands of articles have been published to tackle this crisis and many of these have indicated that high air pollution levels may be a contributing factor to high outbreak rates of COVID-19. Atmospheric pollutants, indeed, producing oxidative stress, inflammation, immuno-unbalance, and systemic coagulation, may be a possible significant co-factor of further damage, rendering the body prone to infections by a variety of pathogens, including viruses. Spermatozoa are extremely responsive to prooxidative effects produced by environmental pollutants and may serve as a powerful alert that signals the extent that environmental pressure, in a specific area, is doing damage to humans. In order to improve our current knowledge on this topic, this review article summarizes the relevant current observations emphasizing the weight that environmental pollution has on the sensitivity of a given population to several diseases and how semen quality, may be a potential indicator of sensitivity for virus insults (including SARS-CoV-2) in high polluted areas, and help to predict the risk for harmful effects of the SARS-CoV-2 epidemic. In addition, this review focused on the potential routes of virus transmission that may represent a population health risk and also identified the areas of critical importance that require urgent research to assess and manage the COVID-19 outbreak.

## Introduction

Coronavirus disease 2019 (COVID-19) due to a new beta-coronavirus severe acute respiratory syndrome coronavirus 2 (SARS-CoV-2) has so far affected almost all countries releasing international panic and alarm. At the time of writing, there have been approximately 120,000,000 confirmed cases and 266 million deaths worldwide. SARS-CoV-2 was first observed in Wuhan, China, in December 2019. However, some reports refer to previous detection of the virus in clinical samples and wastewater in many Western countries (Deslandes et al. 2020; Chavarria-Miró et al. 2020). The virus is very strong and is able to persist in the environment for a relatively long time and preserves its infectivity during this period. Many researchers have discussed the reasons why coronavirus (COVID-19) has spread, persists, and has high contagiousness. An increasing number of studies demonstrate a tight association between chronic exposure to some air pollutants and the transmission and severity of the effects caused by infection of the SARS-CoV-2 virus (Domingo and Rovira 2020). In particular, recent papers strongly associated the exposure to PM2.5 to deaths caused by COVID-19. In addition, the association of ambient air pollutants and meteorological variables with the incidence of COVID-19 has also proposed (Jiang et al. 2020). The stability of SARS-CoV-2 under different environmental conditions supports the hypothesis that air pollution may promote the chain of human-to-human transmission of infection, particularly in situations of high crowding, COVID-19 severity and related risk of death (Domingo and Rovira 2020). In addition, chronic exposure to PM2.5 not only induces inflammation to the alveolar district but also oxidative stress, facilitating the pathogen virulence (Ghio et al. 2012; Li et al. 2019; Tsai et al. 2019; Caso et al. 2020). Recently, human semen is not only considered an early biomarker for monitoring the impact of adverse environmental exposures (Nordkap et al. 2012; Bergamo et al. 2016) but also reflects individuals’ general health condition. In fact, a link between semen quality and the onset of chronic diseases, with male infertility serving as an early predictor of future hospitalization and overall mortality (Jensen et al. 2009; Eisenberg et al. 2015; Pisarska 2017; Choy and Eisenberg 2018).

This review focuses on the evidence that correlates the degree and type of pollution with the increased susceptibility of many countries to this pandemic and proposes human semen as an early marker of the environmental health and of the general health of individuals. These evidences could be useful to suggest an activation of a control policy based on the evaluation of the reproductive health of a given population mainly for those areas at high environmental impact in which already a reduction in fertility or alterations in the spermiogram parameters has been found.

## Air pollution and COVID-19

**Fig. 1:**
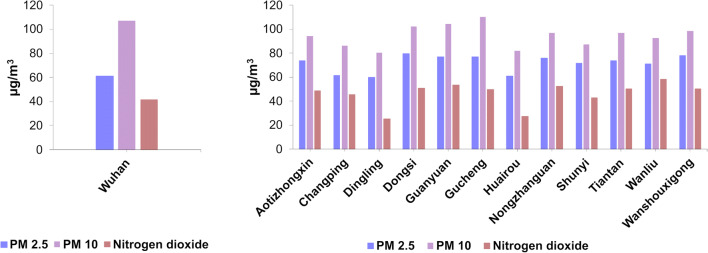
Amount of PM2.5; PM10 and nitrogen dioxide in Wuhan (left) and in other cities of China (right) (data from https://www.kaggle.com/)

Evaluating if long-term exposure to air pollution enhances severity from COVID-19 health outcomes is essential to public health. Indeed, many works indicate that air pollution is an influencing cofactor for the enhanced risk of COVID-19 incidence and mortality (Karan et al. 2020; Fattorini and Regoli 2020; Pozzer et al. 2020; Holme et al. 2020). It is well known that air pollution rates are higher in the winter period, and the cities where COVID-19 had the hardest impact are the one with the annual average PM10, PM2.5, and NO2 above the WHO recommended values of 20 μg/m^3^, 10 μg/m^3^, and 40 μg/m^3^ respectively, like in Wuhan and in other Chinese cities (https://www.kaggle.com/) (Beloconi et al. 2018; Wu et al. 2020b; Pansini and Fornacca 2020a; Paital and Agrawal 2020) (Fig. 1).

Specifically, in Wuhan, a higher mortality rate (CFR) of COVID-19 was also found to be related to the increase in air particulate matter with a diameter of 10 μm (PM10) or less (PM2.5) after adjusting for humidity and temperature (Yao et al. 2020). It is noteworthy that already at the time of the 2003 SARS-CoV-1 infection in China, the CFR in the most polluted areas resulted twice as high in comparison with the least polluted ones (Cui et al. 2003). In addition, in a recent preprint, analyzing China, Italy, and the USA, a significant association was shown between elevated levels of PM2.5, carbon monoxide (CO), nitrogen dioxide (NO2), and COVID-19 diffusion and mortality (Pansini and Fornacca 2020b). Two very recent papers strongly associate PM2.5 exposure with deaths caused by COVID-19. The first, published by Wu et al. (2020a), shows that the highest rates of COVID deaths ascribed to human-caused exposure to particulates matter were in this order: highest in East Asia (~35%), Central Europe (~25%) and Eastern USA (~25%) (Pozzer et al. 2020). These world regions exhibit very high levels of fossil fuel utilization (Pozzer et al. 2020). A second paper instead reports preliminary investigations of this question in the USA, and shows that higher PM2.5 exposures were positively linked to higher COVID-19 mortality rates at the county level after taking into account many area-level confounders. Specifically, in this latter study, a 1 μg/m3 increase in long-term average PM2.5 was related with a statistically significant 11% increase in COVID-19 mortality in the county (Wu et al. 2020a). Numerous further studies are in line with these findings. For example, a study performed in Italy indicated that more than 75% of infected individuals and about 81% of deaths in the first wave of the COVID-19 pandemic in Italy happened in industrialized regions with high levels of air pollution (Skirienė and Stasiškienė 2021). More specifically, it was observed that infected people were higher in those cities that exceeded the set limits for PM10 or ozone for 100-plus days per year, in those cities situated in inland areas (i.e., away from the coast), in cities with low average wind speeds, and in cities with lower average temperatures. In these cities, there was a substantial difference in the average number of people infected in April 2020—during the first wave of the COVID-19 pandemic, which was more than three times as high when compared with the relative number in cities with low levels of air pollution (Coccia 2021). As a matter of fact, the northern Italian regions most impacted by COVID-19 were also the ones presenting the highest PM10 and PM2.5 levels in Europe (Martelletti and Martelletti 2020; Conticini et al. 2020; Fattorini and Regoli 2020).

COVID-19 first spread to China, South Korea, and Iran, and then to Italy before the rest of Europe and the eastern USA, between latitude 30° and 50° North, in the winter period (precisely from December 2019 to April 2020), just when meteorological conditions, such as low temperature (between 5 and 11 °C) and low specific and absolute humidity of 3-6 g/kg and 4-7 g/m^3^ respectively, were propitious for the spread of a respiratory virus (Sajadi et al. 2020).

**Fig. 2:**
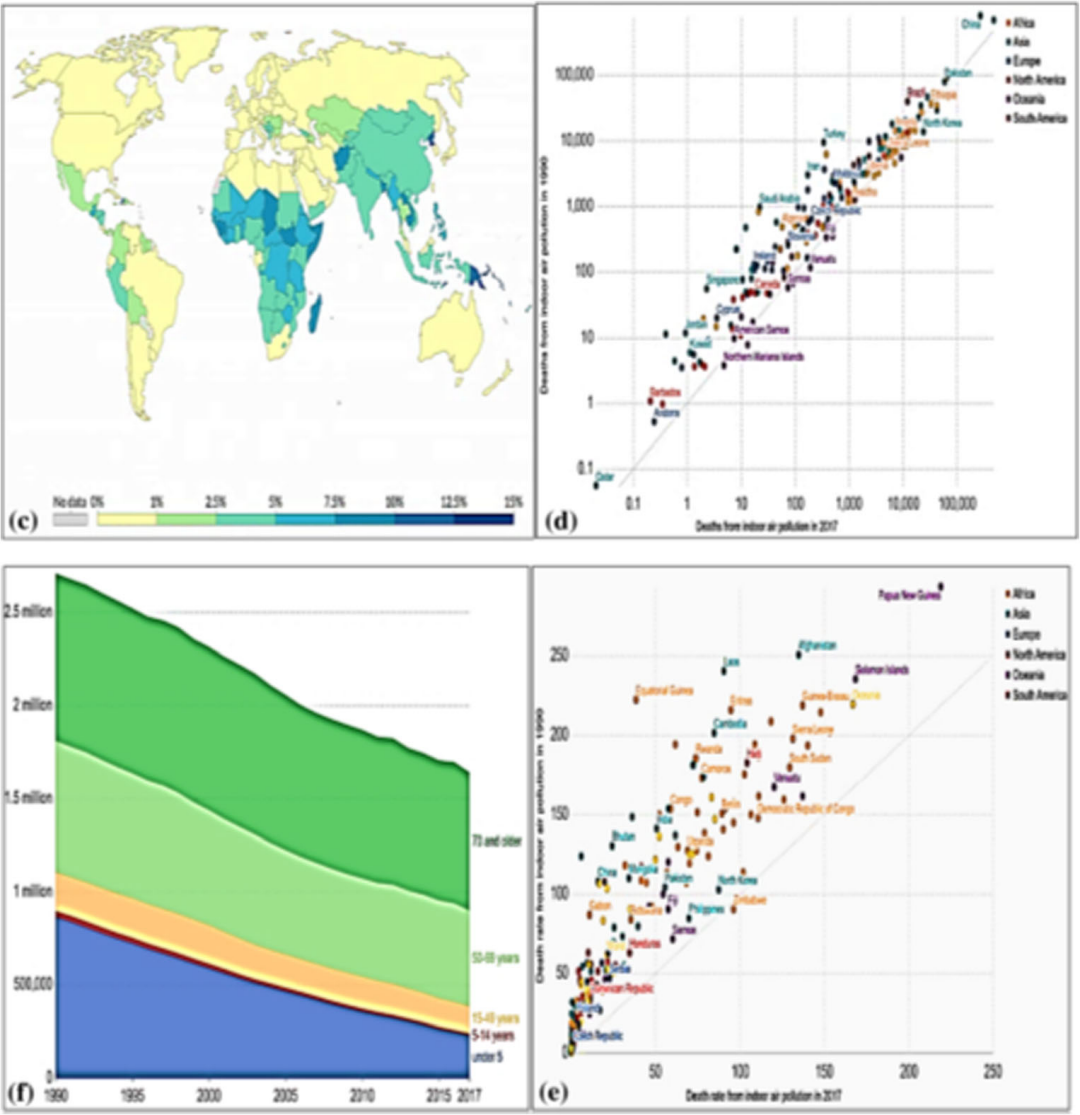
**c** Death mediated by indoor and outdoor air pollution worldwide. An air pollution color intensity illustrating the global death rate produced by indoor air pollution. The percentage displays the rate of death due to indoor air pollution in comparison to other diseases. **d** The number of indoor air pollution deaths in 1990 compared to 2017. **e** Death from indoor air pollution age-dependent. Older people over 69 are at greater risk for indoor air pollution. **f** Rate of indoor air pollution per 100,000 people in 1990 compared to 2017 country distribution. Increasing indoor air pollution is a global concern (Source Institute for Health Metrics and Evaluation 2020 under creative common license attribution). Panels **c**, **d**, **f**, and **e** were reused from Fig. 3 of Paital B, Agrawal PK. Air pollution by NO2 and PM2.5 explains COVID-19 infection severity by overexpression of angiotensin-converting enzyme 2 in respiratory cells: a review. Environ Chem Lett. 2020 Sep 18:1-18. doi: 10.1007/s10311-020-01091-w obtained with License Number 4971970782195

Moreover, a retrospective study on the link between air pollutants and meteorological variables with the COVID-19 occurrence demonstrated the relationship of some meteorological parameters, especially relative humidity with PM2.5 and COVID-19 in three Chinese cities: Wuhan, Xiaogan, and Huanggang (Jiang et al. 2020). Nevertheless, it is still unclear how the SARS-CoV-2 virus can propagate via PM2.5 particles (Jiang et al. 2020) although indoor air fine particulate matter (Fig. 2c, d, f, e) with a diameter of ≤ 2.5 μm (PM2.5) has been reputed to be implicated as a transport agent for the virus (Nor et al. 2021).

## Pollution and susceptibility to viral insults

The World Health Organization (WHO) reports that approximately a quarter of diseases are caused by exposure to environmental pollutants over time (GBD 2015 Risk Factors Collaborators 2016). These diseases include cardiovascular and chronic degenerative disorders, early deaths, and reproductive impairments (Cohen et al. 2005; Ji and Zhao 2015; GBD 2015 Risk Factors Collaborators 2016) together with lifestyle, as mentioned in the European Code against Cancer (Anderson et al. 2015). Furthermore, environmental pollutants have the potential to enhance susceptibility to non-communicable disease (NCD). The consequence of all this is a marked decline in the organism’s defenses, which is probably attributable in part to trans-generational effects that may explain some of the worldwide disease burden of illness that comprises not only NCDs but also communicable diseases (Curley et al. 2011; Soubry et al. 2014; Braun and Champagne 2014; Anderson et al. 2015; Zhao et al. 2017; Horan et al. 2017; Bianco-Miotto et al. 2017; Suzuki 2018; Soubry 2018a, b; Xavier et al. 2019; Heindel 2019; Post et al. 2019). In addition, transgenerational effects have also been shown to include a reduction in the ability to defend against viral pathogens (Post et al. 2019). In the mouse model, recent studies have shown that chronic exposure to PM2.5 results in increased expression of the angiotensin-converting enzyme (Lin et al. 2018).

Besides, the chronic exposure to PM2.5 not only leads to inflammation in the alveolar district but also, after passing the alveolar barrier, arrives to the blood and then to the peripheral tissues, and induces oxidative stress both directly and via the response of the host to the chemical insult. This in turn induces inflammasome activation and, in particular, NLRP3, affecting the maturation and secretion of cytokines such as IL-1 beta and IL-18, both involved in the systemic inflammatory syndrome. Such conditions would promote the virulence of the pathogen, which includes vascular leakage and coagulopathy (Ghio et al. 2012; Li et al. 2019; Tsai et al. 2019; Caso et al. 2020).

The ability of SARS-CoV-2 to trigger a rapid autoimmune dysregulation process, results in the generation of a significant storm of cytokines, principally TNF-α, IL-6, and IL-1β, IL-17, IL-18, in individuals who are genetically predisposed (Masters et al. 2009). This process may become even more significant when preexisting environmental factors have previously altered mechanisms regulating cytokine release and/or also when certain polymorphisms for IL-6 are present, such as among specific populations or ethnic groups that would render them more prone to virus complications (El-Maadawy et al. 2019). To be precise, all populations are susceptible to COVID-19, but the people most susceptible to complications of this disease turn out to be the elderly, individuals with chronic diseases, or low immunity, pregnant women, and newborns (Working Group for the Prevention and Control of Neonatal 2019-nCoV Infection in the Perinatal Period of the Editorial Committee of Chinese Journal of Contemporary Pediatrics 2020; Huang et al. 2020; Chen et al. 2020).

In addition to this, it should be added that, air pollutants represent a potential co-factor of major damage because they are capable of inducing oxidative stress, inflammatory processes, immune imbalance, and coagulation at the systemic level. All these effects, consequently, make the organism susceptible to complications from pathogens, including SARS-CoV-2 (Glencross et al. 2020).

**Fig. 3:**
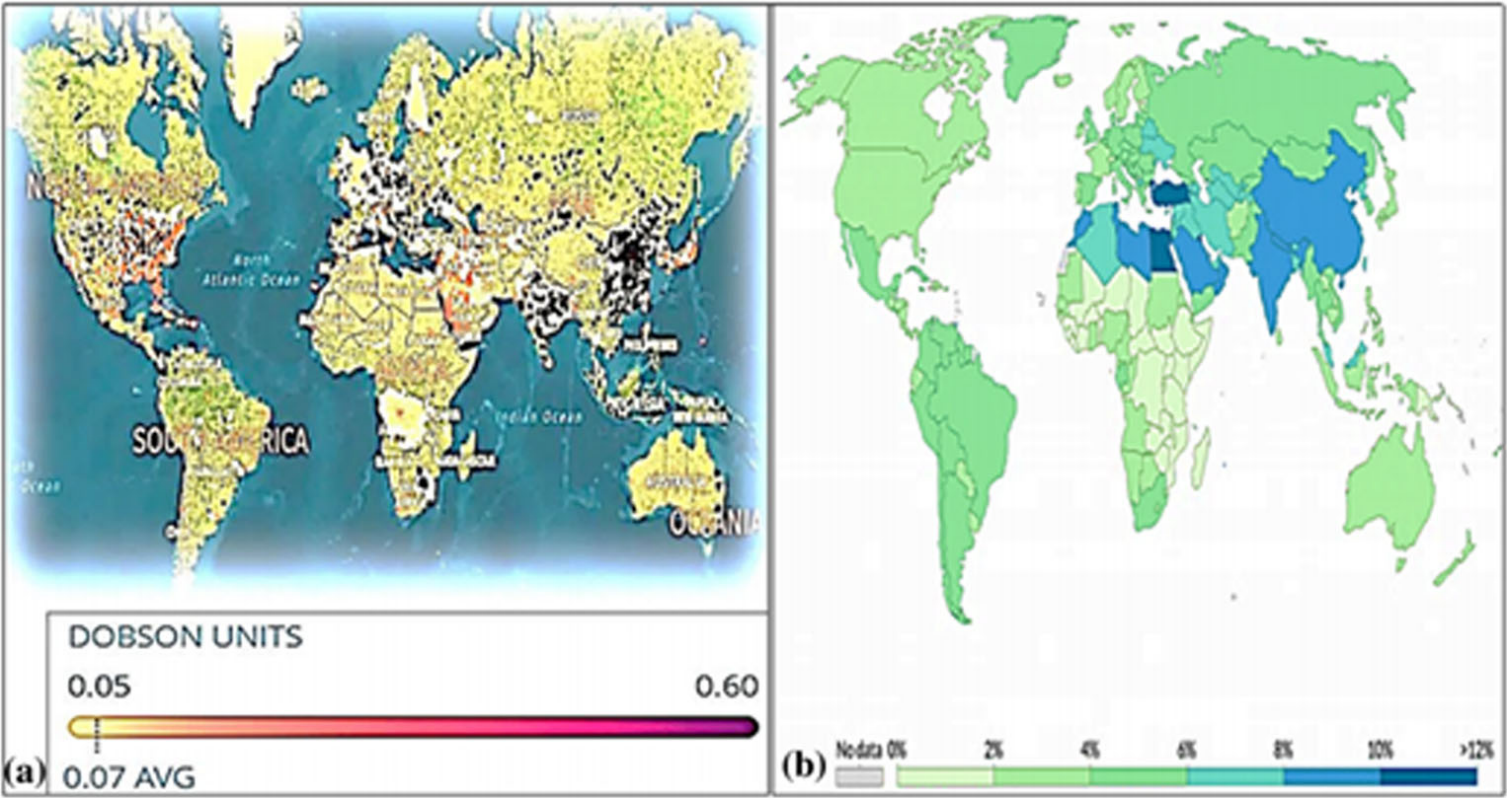
Panel **a** shows the world map of tropospheric NO2 concentrations from the Sentinel-5P satellite (2018). The values were measured daily for 90 days, from June to August 2018 (Chow 2020; Environmental Protection Agency, 2020, Myllyvirta and Howard 2020) (**a**). Panel **b** shows instead the world global death rate (%) by air pollution map in comparison with other diseases. Color intensity illustrating the global mortality rate due to air pollution (**b**). Panels **a** and **b** were reused from Fig. 3 of Paital B, Agrawal PK. Air pollution by NO2 and PM2.5 explains COVID-19 infection severity by overexpression of angiotensin-converting enzyme 2 in respiratory cells: a review. Environ Chem Lett. 2020 Sep 18:1-18. doi: 10.1007/s10311-020-01091-w obtained with License Number 4971970782195

This situation is still more real in those areas of the world where a very poor air quality, reducing the antioxidant, and immune defenses of the body, could promote viral contagion and/or increased virulence. This is so true that, as well described by the Paital and Agrawal 2020 studies, the overall mortality rate (%) from air pollution is higher than for other diseases as can be well understood from Fig. 3 which shows the world map of tropospheric NO2 concentrations from the Sentinel-5P satellite (2018) (Fig. 3a) and the world map of global mortality rates (%) from air pollution relative to other diseases (Fig. 3b) (Paital and Agrawal 2020).

Interestingly, NO2 is also potent in inducing upregulation of the ACE-2 enzyme and a positive correlation between NO2 and COVID-19 has been observed in countries such as Europe: France, Italy, Spain, and Germany (Ogen 2020), coherently with Fig. 3a. SARS-CoV-2 can interact with the renin-angiotensin-aldosterone system (RAAS) through ACE-2 (Ogen 2020). The RAAS is a hormonal system that controls blood pressure and fluid and electrolyte balance, and also systemic vascular resistance (Fountain and Lappin 2020).

## Sperm decline in polluted areas

**Fig. 4:**
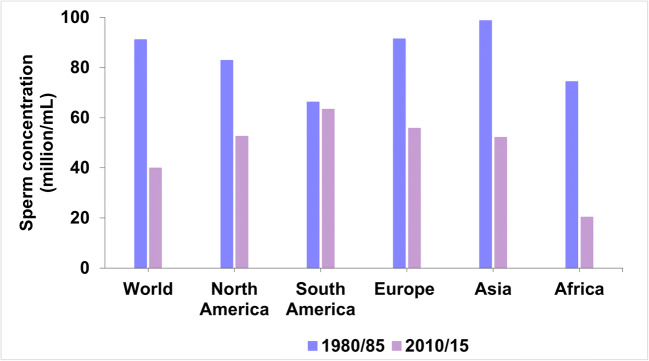
The figure shows the decrease of the sperm concentration (million/mL) observed in North America, South America, Europe, Asia, and Africa from 1980/85 to 2010/15 (Agarwal et al. 2015). The mean decrease of the sperm concentration in the word in those years has been about 57%

Several data have indicated that there has been a decline in sperm parameters in several areas of the world over the past four decades. This decline has been particularly notable in those areas of strong industrial development that had high levels of air pollution (Mann et al. 2020). This is well described in Fig. 4 showing the decline in sperm concentration (million/mL) that occurred in North America, South America, Europe, Asia, and Africa, from 1980/85 to 2010/15 (Agarwal et al. 2015). As shown in the figure, the average decrease in sperm concentration worldwide during those years was about 57%.

In addition, there is a systematic review and meta-regression analysis in the literature reporting a 59.3% decline in total sperm count in Europe, the USA, Canada, and New Zealand considering the years from 1971 to 2011 (Levine et al. 2017). In Asia, over the past 20 years, there has been a 20% increase in the infertility rate of Iranian men while in China, a study that considered a total of 30,636 young donors, showed that sperm concentration had decreased from 68 × 106/mL to 47 × 106/mL, while the percentage of spermatozoa presenting normal morphology was reduced from 31.8 to 10.8% (Safarinejad 2008; Huang et al. 2017). A decline in sperm count has also been recorded in Brazil, where, over the past 23 years, there has been an average sperm reduction of 0.24 million/mL per year (Siqueira et al. 2020).

The male reproductive system is particularly sensitive to environmental pollutants and chronic exposure to high levels of PM10, PM2.5, and to individual air pollutants such as NO2 and sulfur dioxide (SO2) negatively impacts sperm count, motility, and testicular volume (Zhang et al. 2020). The mechanisms of damage to spermatogenesis caused by environmental agents are still largely unknown, although the imbalance of oxygen radical species (ROS) and the resulting oxidative stress may represent the common denominator by which pollutants alter seminal parameters such as sperm count, motility, morphology, and especially the integrity of sperm DNA (Bosco et al. 2018; Jurewicz et al. 2018; Nowicka-Bauer and Nixon 2020). In addition, a very interesting review has recently been published that provides the current state of knowledge on the impact of a wide range of environmental stressors on several parameters used to estimate and assess gamete quality in humans and in the main animal models that are used for experimental research. In particular, this review examines the effects of metals, biocides, herbicides, nanoparticles, plastics, temperature rise, ocean acidification, air pollution, and lifestyle on the physiological parameters that underlie gamete fertilization competence. This review strongly emphasizes the concept that environmental stressors pose a serious threat to gamete quality with reproductive disorders and failure of the living organism (Gallo et al. 2020). Moreover, compared to oocytes, spermatozoa are very sensitive to the pro-oxidant effects of environmental pollutants. The reason for this lies in the fact that these cells have a limited volume of cytoplasmic space, with less antioxidant defense, and in addition the lipids of the sperm membrane are the target of ROS (Aitken et al. 2016). Male gametes are also the most sensitive cells to the accumulation of damaged DNA so in our research we have investigated to try to understand the reasons. Through molecular investigations, we have recently shown that sperm nuclear basic proteins (SNBP) from samples of men living in polluted areas have a novel and unexpected behavior, being involved in oxidative DNA damage (Lettieri et al. 2020a). Furthermore, we have also reported preliminary results that appear to indicate transgenerational effects of pollutants on molecular alterations in SNBPs in humans living in polluted areas (Lettieri et al. 2020b). These data are in line with those obtained in mice, in which has been demonstrated that the susceptibility to certain pollutants increases across generations (Horan et al. 2017). The negative trend in sperm quality in conjunction with annual high average levels of PM10, PM2.5, and NO2, could suggest that sperm decline may be the first clinical sign of environmental pressure and that semen quality could be a potential indicator of susceptibility to insults in polluted areas including viral infections as described in the following section.

## Human semen as environmental and health marker

The sperm chromatin structure is often impaired, largely due to oxidative damage. As a matter of fact, many pollutants, including heavy metals, cause molecular genotoxic effects and induce alterations in chromatin remodeling. Specifically, multiple histone sites, depending on heavy metal coordination within the nucleosome, may represent active sources of ROS generated in DNA proximity (Mohideen et al. 2010). Some marine organisms and plants are extensively used as bioindicators of environmental pollution (Basile et al. 2017; Maresca et al. 2018, 2020). In this regard, we have recently provided new insights on some metals toxicity mechanisms in marine organisms (Piscopo et al. 2018a, b, c; De Guglielmo et al. 2019; Lettieri et al. 2019).

Our knowledge regarding the molecular mechanisms underlying the toxicity of metals on the reproductive health of marine organisms, gained through studies of these organisms, has been useful in understanding the role of pollutants on human reproductive health. Our studies on individuals living in polluted areas demonstrated that also human semen could be considered an ideal early sentinel with a double function: environmental and human health (Lettieri et al. 2020a, b). Other authors besides us point to human semen as a “sentinel biomarker” of subclinical biological effect suitable for monitoring the impact of adverse environmental exposures (Nordkap et al. 2012; Bergamo et al. 2016; Montano et al. 2017).

Semen quality has also been found to reflect individuals’ general health condition, as recent studies had shown an association between semen quality and the onset of chronic diseases, with male infertility being a predictor of future hospitalization and overall mortality (Jensen et al. 2009; Eisenberg et al. 2015; Pisarska 2017; Choy and Eisenberg 2018).

We assume that these observations could support the comprehension of the dynamics that may have been involved in the facilitation of COVID-19 severity in polluted areas. As an early and responsive environmental and health marker, semen quality could be used as a key indicator of sensitivity to environmental health hazards in the general population and be utilized for health risk management, innovative prevention programs, and health surveillance, especially in heavily polluted areas. However, although factors such as high population density, climatic characteristics, age, comorbidity, different health systems’ ability to respond to the pandemic, and the prevention policies employed in different countries presently play a pivotal role, we should not underestimate the possible facilitatory role of pollution in augmenting the risk of contracting COVID-19 for people living in areas with greater environmental impact.

In addition, there is also evidence that these same areas have a greater incidence of NCD and male infertility due to a complex interrelationship of chronic chemical and physical exposure factors, in conjunction with the contribution of lifestyle behavioral risk factors and the genetic background of individuals. In addition, exposure to heavy metals such as As, Cd, Hg, and Pb has also been shown to be linked to respiratory dysfunction and disease (Skalny et al. 2021). Of course, this evidence is supportive in explaining the role of heavy metal exposure in compromised mucociliary clearance, diminished barrier function, respiratory tract inflammation, oxidative stress, and apoptosis. Moreover, an association has also been provided between heavy metal exposure and the severity of viral diseases, including influenza and respiratory syncytial virus (Skalny et al. 2021). The latter may be considered a result of the adverse effects of metal exposure on adaptive immunity. Thus, early signs of damage to organ-sentinel systems such as the male reproductive system and the decline in human sperm quality observed in recent decades may hint at how relevant environmental pressures may be and how increasingly unsustainable it is becoming. Finally, through the use of human semen, as an early sentinel of environmental and general human health status, we could have an opportunity to know the population health status in a given environmental context and go to predict both the susceptibility to the virus impact and the medium and long-term negative effects on human health.

## Conclusion

A robust body of experimental evidence suggests that pollution should be recognized as an important cofactor for increased transmission and severity of SARS-CoV-2 infection outcomes and mortality. It was therefore interesting to learn of the significant reduction in NO2, CO2, and O3 as a result of the decrease in human activities during the SARS-CoV-2 outbreak in northeast China (epicenter of the COVID-19 pandemic onset) known to be affected by this massive increase in carbon emissions due mostly to transport (Dutheil et al. 2020a, b; Wang et al. 2020). Thus, in the extremely dramatic context of the SARS-CoV-2 pandemic, this overall decrease of atmospheric pollution has offered a very strong signal for the conservation of our environment and some related cardiovascular and respiratory diseases (Guan et al. 2016; Cramer et al. 2020). We also believe that semen quality used as an early environmental and health marker could help policy makers to promptly intervene in areas with significant environmental criticality to abate air, water, and soil pollution with an integrated One Health approach, where information sharing among diverse and key professionals (clinicians, biologists, chemists, virologists, veterinarians, economists, epidemiologists) could be successful in establishing a systemic approach that could be efficient and beneficial on a global scale (Mackenzie and Jeggo 2019). Above all, it is essential to evaluate the effectiveness of the measures adopted to safeguard the community’s health and also its social and productive organization, with the objective of avoiding, or at least limiting, the rapid and destructive spread of future viruses.

## Data Availability

The authors declare to acquire the permission for use of Figs. 2 and 3. Air pollution by NO2 and PM2.5 explains COVID-19 infection severity by overexpression of angiotensin-converting enzyme 2 in respiratory cells: a review. Environ Chem Lett. 2020 Sep 18:1-18. doi: 10.1007/s10311-020-01091-w obtained with License Number 4971970782195.
